# Wireless Sensor Platform for Cultural Heritage Monitoring and Modeling System

**DOI:** 10.3390/s17091998

**Published:** 2017-08-31

**Authors:** Levente J. Klein, Sergio A. Bermudez, Alejandro G. Schrott, Masahiko Tsukada, Paolo Dionisi-Vici, Lucretia Kargere, Fernando Marianno, Hendrik F. Hamann, Vanessa López, Marco Leona

**Affiliations:** 1 IBM Research, Thomas J. Watson Center, Yorktown Heights, New York, NY 10598, USA; sergio.bermudez@me.com (S.A.B.); ag.schrott2015@gmail.com (A.G.S.); fjmarian@us.ibm.com (F.M.); hendrikh@us.ibm.com (H.F.H.); lopezva@us.ibm.com (V.L.); 2 Department of Scientific Research, The Metropolitan Museum of Art, New York, NY 10028, USA; m.tsukada.tnm@nifty.com (M.T.); paolo.dionisivici@gmail.com (P.D.-V.); marco.leona@metmuseum.org (M.L.); 3 Objects Conservation Department, The Metropolitan Museum of Art, New York, NY 10028, USA; lucretia.kargere@metmuseum.org

**Keywords:** micro environmental monitoring, wireless sensor network, air quality monitoring, corrosion, computational fluid dynamics

## Abstract

Results from three years of continuous monitoring of environmental conditions using a wireless sensor platform installed at The Cloisters, the medieval branch of the New York Metropolitan Museum of Art, are presented. The platform comprises more than 200 sensors that were distributed in five galleries to assess temperature and air flow and to quantify microclimate changes using physics-based and statistical models. The wireless sensor network data shows a very stable environment within the galleries, while the dense monitoring enables localized monitoring of subtle changes in air quality trends and impact of visitors on the microclimate conditions. The high spatial and temporal resolution data serves as a baseline study to understand the impact of visitors and building operations on the long-term preservation of art objects.

## 1. Introduction

Among public buildings, museums represent a special case with respect to environmental management. Not only do they have to be comfortable to humans, but must also provide conditions that adhere to more stricter environmental standards in order to preserve the cultural heritage objects in their custody [1]. There is an ongoing discussion regarding the general applicability of these standards and there are studies focused in assessing the possibility of establishing wider windows of temperature/humidity conditions tuned more specifically to distinct kind of objects [2,3,4]. This is a very ambitious, although necessary endeavor, since the confluence of higher energy costs and the effects of climate change will be affecting the way buildings are constructed, refurbished and managed [5].

Depending on the building, there may be competing effects from mechanical cooling/heating and the location, age, and construction material of the buildings. The outdoor conditions may play a role in changing the indoor conditions, sun may warm some walls or windows during daytime while in the winter time excessive cooling may occur that could result in condensation. Additionally, as doors are opened for visiting groups of people, drafts may propagate across multiple rooms and potentially connect the indoor to the outdoor environment. The inherent dynamic changes in the environment are hard to capture with a limited number of sensors and there is a need to better understand how these fluctuations may change daily or seasonally. Furthermore, architectural design and Heating, Ventilation and Air Conditioning (HVAC) operation can lead to the formation of microclimates that are distinct from their surroundings. Any approach to change museum environmental conditions based on existing standards will have to be based on longer term and robust results to be accepted both by private art collectors/donors of art and also by insurance companies [6].

Towards that goal, the first necessary step is to have information of the dynamics of the environmental fluctuations inside the buildings in a comprehensive way throughout long periods of time to ascertain seasonal, architectural and visitor’s impact on the local micro-environment [1,5,7,8,9].

Typically, HVAC systems are designed to operate in a quasi-steady state with feedback to the established operational settings relying generally on sensors located at the supply and return vents of the rooms. Closed control loops may be employed to maintain temperature or relative humidity constant with minor fluctuation around the set-points. However, using a single or a handful of sensors may not be enough to predict in a comprehensive way the micro-environmental fluctuations. Generally, the physical environments of art exhibitions are intrinsically complex, responding to prior architectural design (churches, castles, ancient buildings) and many times cannot be modified. Thus, new platforms [7,10,11,12,13,14,15] aiming at providing a denser mapping of environmental conditions are desirable to understand how new environmental standards may affect current collections [1,16,17]. Furthermore, a correlation of this local dynamic environmental information with the response of the art pieces should be a necessary part of the endeavor [18,19,20]. This correlation is a very laborious task which needs to encompass not only measuring the response of suitable test vehicles in a laboratory coupled with modeling [21], but also the assessment of the validity of such models by directly monitoring the actual works of art. The latter presents its own set of difficulties since often it has to overcome curatorial distrust regarding the appearance, position and possible damage to the object by sensors.

Wireless sensor networks have evolved to fulfill the application of dense and precise measurement of environmental conditions in real time [10,11,22]. Such precise measurement can be done even under dynamic conditions, when air is mixed from the top to the bottom of each room, and temperature and air humidity are acquired at the same air pressure (e.g., Mollier diagrams) [23]. The advantage of wireless sensors is the ability to combine sensing, analytics and control while being easily deployable in existing galleries and being easily repositioned as required by changing exhibitions. The wireless sensor network eliminates the traditional need of extension cables to provide power and capture sensor data, while the sensors’ small size allows them to be placed nearby art objects without being visually obtrusive. The second advantage of wireless sensing is the embedded computational capability that enables multiple sensing points to be attached to the same radio-transmitter, thus reducing the cost per sensing point.

Beyond the traditional sensor signal monitoring of temperature, relative humidity, air flow or pressure, recent trends are to create platforms that would include additional sensors like noise, illumination, and air quality sensing. These new sensing platforms are going beyond simple environmental monitoring, as they try to quantify in real time the impact of fluctuating environmental conditions on exhibited objects [18,19,20,24,25]. One other possibility is a control mechanism where optimization of the environment can be implemented based on constrains (like optimizing the number of people in galleries such that temperature and humidity will not fluctuate outside a desired range). Recently wireless sensor networks were applied to adjust illuminations in galleries and demonstrating that a wireless node can act simultaneously as a sensor and actuator [26]. Dense sensor networks are enabling a holistic understanding of how the local microenvironment are dynamically changing in the museum.

Beyond environmental monitoring, there is a sustained activity in optimizing wireless communications and ensure that data integrity, security and dynamic sampling can be easily integrated. Many of the current research areas [27,28,29,30,31] focus on energy efficient routing, congestion control, congestion avoidance, extending connectivity and expanding coverage. All these advancements can expand current monitoring platforms capabilities by enabling dynamic data sampling and modeling based on automatically triggered events.

This paper describes the sensing platform installed at The Cloisters, the medieval branch of the New York Metropolitan Museum of Art, and presents examples of the kind of local environmental information obtained as well as physical analytical modeling of the sensed data as correlated with outside conditions and visitor flow. In this project, the Low-power Mote Technology (LMT) by IBM (Armonk, NY, USA), along with IBM’s Measurement and Management Technologies (MMT) solution, were utilized. The MMT platform supports sensor anomaly detection, real time data processing and thresholding. Furthermore, physical based models can be used to interpolate values from the collected sensor data at the sensing points [32]. LMT supports several types of sensors, for the various measurements of interest in a museum and their characteristics will be discussed in the corresponding sections.

**Figure 1:**
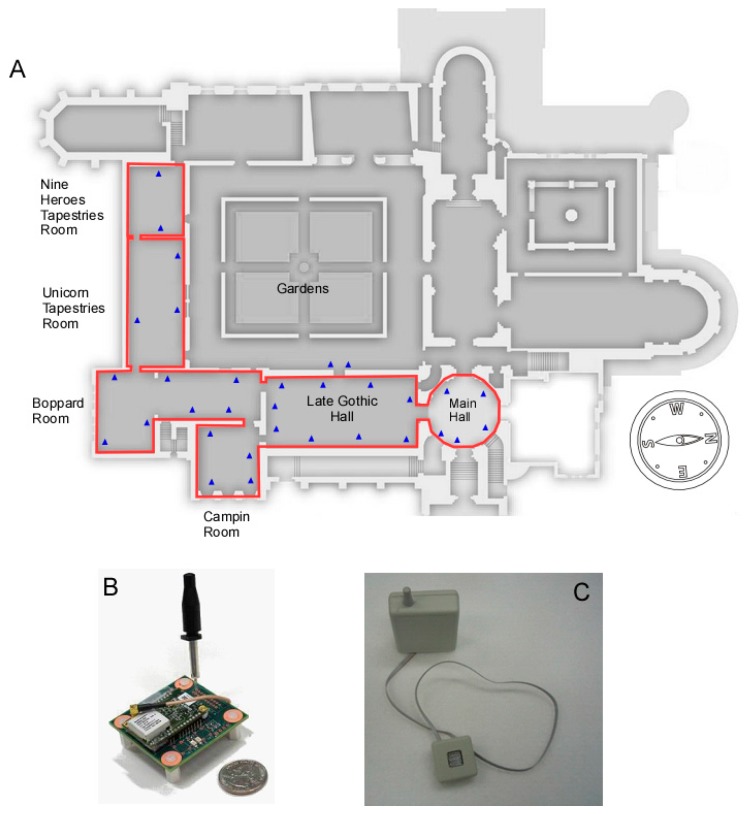
(**A**) Layout of the 5 galleries at the Metropolitan Museum of Art, The Cloisters, where more than 200 wireless sensors were distributed. Picture of a single mote (**B**) containing the communication radio and sensors and a corrosion sensor (**C**) used for air quality monitoring.

The Cloisters is characterized by distinct historical and architectural features. For stylistic reasons, the design of The Cloisters centers on a massive masonry construction. Like many medieval castles in Europe, the building was also meant to conserve heat during winter and reduce thermal gain during the summer. However, the composite nature of the building, with medieval stone windows inserted in a modern matrix, and galleries opened to gardens, somewhat limits the quality of the envelope. At its inception in 1938, The Cloisters were heated by the standards of the day with oil heat, but no provision was made for air conditioning, or full humidity control. At the time, architects wanted to minimize the use of modern mechanical systems, to create an intimate relationship between visitors and the collection. Reducing their size to the utmost reasonable limit, the visual appeal of heating grilles was just as, if not more important, than their efficiency. The system provided some humidity in the winter, so the relative humidity (RH) values would not drop below 30%, but no dehumidification was performed throughout the year. The relative humidity ranged between 50% and 90% during the summer, falling down to 30% in the winter, with temperatures in summer frequently above 27 °C, and wide fluctuations throughout, especially in the spring and in the fall. It is mentioned in the museum archive that in order to avoid condensation on the stained glass collection, the entire air washing system was turned off to provide humidity when the outside air fell below −12 °C [21]. Despite the introduction of an air washing systems, pollutants caused by oil heating, mostly chlorides and sulfates, are mentioned in early museum reports. In 1969, a “demineralizer” was introduced in the Campin and the Tapestry rooms (see Figure 1A for a floor plan), along with a localized controlled environment. In 1987, the Treasury room was equipped with a similar system [33]. It is only in 2005 that the long deferred task of installing a full-scale climate control system throughout the galleries was finally realized at The Cloisters. However, some specific issues are still being addressed, such as the problem of condensation on windows.

Condensation is quite common in temperate climates due to the thermal gradients that form across the window panes when the indoor/outdoor weather conditions are significantly different. In general, glass is considered to be a relatively stable material, but it can deteriorate in inadequate environmental conditions, depending on the chemical composition of the materials used in its production. For example, by prolonged contacts with liquid water or highly moist air in acidic environments, alkaline and alkaline earth metal ions in glass are exchanged by hydrogen containing ions from water, and are leached out to the surface, forming a hydrated layer. In alkaline environments hydroxide ions from water attack the silicate network of glass, thus causing the glass dissolution [34]. Glass rich in potassium and calcium contents, which was often used for medieval stained glass windows, is typically vulnerable to the former alteration process. The formation of a hydrated layer at the surface leads to various degradation phenomena, such as formation of micro cracks, pits, crystalline efflorescence and encrustation. In order to prevent stained glass from those environmentally induced weathering phenomena, the installation of protective glazing [35], which is a transparent sheet material (modern, more stable glass or plastic) placed at the outside of the original window, is widely adopted. Still, even after the installation of protective glazing, air circulation in the interspace between the original window glass and protective glazing is an important factor to prevent the condensation and high humidity [36]. Condensation risk can be assessed using forecasted weather data combined with models that predict heat conduction across glass windows. We discuss such a model that was developed for day ahead prediction of condensation risk during winter months.

Air quality is an important aspect of indoor environmental condition in museum as chemical pollutants may impact metal, wood and textile objects. Most commonly the air quality is assessed using metal coupons (silver, copper, lead) exposed to the environment and quantifying the corrosion product building up on the surface. Air quality standards are developed to quantify the maximum amount of pollutants that can be tolerated in museum environments. In this study corrosion sensors fabricated from silver and copper were deployed in multiple galleries to measure long term trends in air quality change, and the MMT platform calculated in real time the corrosion rate of the sensors based on a sliding window algorithm [37]. While our study demonstrates that the environmental conditions within the galleries are very stable, the dense sensor networks installed in 5 galleries enabled micro environmental studies of visitors’ impact and long term air quality. The data from the dense sensor networks can be used to improve long term microclimate understanding in galleries, provide real time insight into short term fluctuations, and quantify the connection between the indoor and outdoor climates.

## 2. Materials and Methods

### 2.1. Monitoring Technology

There are several monitoring technologies for museums and their common, desirable characteristics is that they should: (1) provide sensitive, accurate and high frequency measurements for an extended period of time and (2) be visually unobtrusive—e.g., no visible probes or wires around art objects. Wireless sensor networks fall within these categories, allowing untethered devices to be powered by batteries, which can be hidden behind works of art [38]. Since our interest is developing accurate microclimate models within a museum, it is necessary to have multiple sensing points in every gallery that facilitate dense monitoring in space and high frequency in time (one data point per minute). As a monitoring technology we use a wireless sensor network platform, where the radios (called motes due to their small form factor) provides automatic data collection. Motes are composed of sensors, a microcontroller, and a radio. Sensor data can be acquired continuously at fixed time intervals or data acquisition can be triggered by external signals that can be used to start the data collection and to determine its duration. One aspect that sets apart our monitoring platform from other platforms [11,12,15] is the integrated analytics and modeling capabilities, beyond data acquisition, that convert raw data measurements into information that can be easily used by curators. Furthermore the power efficient sensors combined with advanced wireless communications allow extended period of operations on batteries without the need for frequent replacement.

The spatial coverage of the sensor deployment is shown in the floor plan in Figure 1A where the motes were placed at different heights in the galleries. For example, in the Late Gothic Hall the sensor placement height ranged from 0.5 m up to 11 m. One driving consideration for sensor placement was the investigation of temperature stratification in galleries and second acquisition of enough sensing points for modeling. The majority of the motes were placed around art objects in close proximity of walls and were concealed to not interfere with the aesthetics of display art objects. The temperature and humidity sensors were positioned to face the gallery and air was allowed to freely flow across the sensor surface.

The data from the wireless sensor network is supplemented with external data sources like outdoor weather and air quality measurements. The real time data stream from motes enables an immediate insight on environmental changes due to visitor flow or change in air quality.

A typical sensor mote, with a default one temperature and one humidity sensor integrated into the processing board, is shown in Figure 1B. Additionally the following sensors can be added to the motes: air quality, visitor presence, and door position. Next we briefly mention the basic characteristics of each sensor and its purpose. All of the sensors use high precision components (some off-the-shelf and some custom-developed at IBM) and are implemented using low power design in order to prolong the mote battery lifetime and thus minimize the number of required maintenance steps.

The temperature transducer is based on a semiconductor diode element (TMP112 with 12-bit resolution, Texas Instrument, Dallas, TX, USA) with an accuracy of 0.5 °C. For example, air temperature measurement is used for evaluating the thermal stratification along the height of a gallery and also slight temperature gradients between different galleries. The relative humidity (RH) sensor is based on a capacitive element (SHT21, 14-bit resolution, Sensirion, Stafa, Switzerland), with an accuracy of ±2%. The RH transducer is useful for evaluating air moisture levels. There are also alternative high precision humidity measurement methods based on capacitance changes like quartz humidity that are characterized by high sensitivity, high temperature stability, immunity to electromagnetic interference and fast response time. The differential sensors and the new switching sensor methods are cost effective while at the same time fulfill the requirement for high precision measurements [39,40].

The dew point was calculated from temperature and relative humidity measurements and is as an indicator of condensation risk on windows, water condensation over painted glass or appearance of condensation on the walls. Dew point measurements are also relevant to understand the acceleration of corrosion rate measurements that relates to air quality consideration within the museum.

All the deployed sensors were adjusted by comparing their data with those of calibrated scientific level temperature and relative humidity sensing devices by exposing them to the same ambient environment. Temperature and relative humidity sensors readings were within the sensors accuracy specification and were compared with calibrated sensors used by curators during the study period. We note, that the long term stability of LMT sensors were validated in a data center environment [41].

The air quality sensor uses a chemically sensitive resistive element to detect pollutant gas concentrations (Figure 1C). The resistive air quality sensor measures the change in the film thickness as gaseous molecules get absorbed on the metal film surface and transform the top layers of the sensor element from metallic to insulating layer. As a way of example, sulfur-bearing gases will get absorbed on the silver surface and transform it into silver sulfide (Ag_2_S). While silver is electrically conductive, Ag_2_S is nonconductive and reduces the physical thickness of the conductive layer increasing the resistance of the metal layer. Similarly, moisture on the top of a copper sensor in combination with sulfur bearing gases will form Cu_2_S and CuO. Both silver and copper thin films are used to determine the rate at which the film thickness is changing. Silver sensors are very sensitive to interaction of sulfur bearing gases (H_2_S, SO_2_) while copper is sensitive to the combined effect of SO_2_ and NO_2_ gases. Other contaminants like CO_2_ will not impact corrosion sensor reading but their minimization is important to maintain visitor comfort in the museum. The resistive signals from the air quality sensors are digitized by a 24-bit resolution analog-to-digital converter. Corrosion sensor reading were validated using side by side comparison with metal coupons whose corrosion byproduct was measured using coulometric reduction [42]. The corrosion rate readings for both techniques are within 10% which is comparable to the accuracy in measuring film thickness in both techniques. Both corrosion measurement techniques were used in the past to create ASHRAE standard recommendation for air quality control in mission critical facilities [43].

Some LMT motes have a movement sensor, which is based on a passive infrared diode. These motes are strategically placed within galleries (i.e., access points, particular art objects of interest) and their measurements help estimate the number of visitors in a given gallery. Automatic measurements of visitors’ paths in a given gallery can be valuable to learn about their cultural interests and also, in general, for space utilizations in order to arrange exhibitions such that crowding can be avoided. Estimating the number of visitors in real-time is also useful for modeling the relationship between human presence impact on microclimate—below we expand on this point.

Finally, based on the architectural design of the museum, direct external air flow is possible through corridors and galleries. To account for these effects, LMT has sensors that monitor the position of several doors—detect when each of the doors is open or closed. The insight provided by a simple reed switch is relevant to use it in the models discussed below—given that the doors are a direct link between the galleries and the outside climate.

### 2.2. Modeling Techniques

Two different types of modeling were implemented, each with its own particular characteristics, which utilize the data produced by our sensing technology. The sensor data serves for both techniques as a set of boundary data.

**Figure 2:**
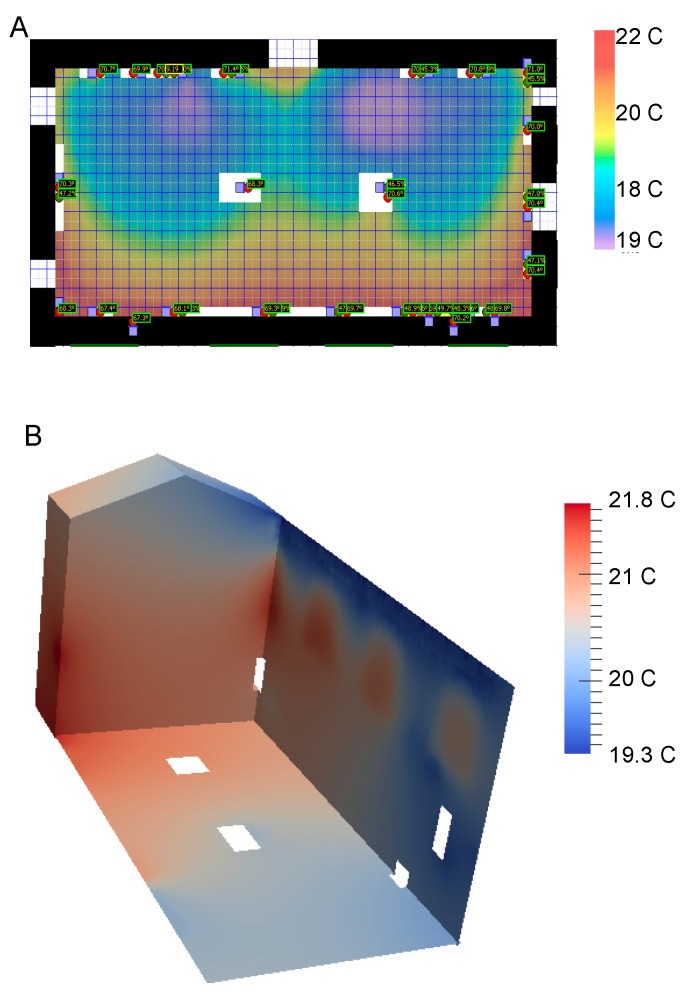
(**A**) Interpolation of the temperatures in Late Gothic Hal at 0.5 m height using sensor readings from the Measurement and Management Technology platform. (**B**) Computational Fluid Dynamics simulations of the temperature on the East/North walls using real time sensor data.

For the first modeling technique an interpolation method is used, where temperature and air moisture are estimated using a smooth bivariate interpolant to the scattered sensor data that is locally a quintic polynomial in two variables [44]. This method is valid in locations where the temperature is smoothly varying on short distances (Figure 2A) but most likely is losing accuracy near air inlet and outlet in galleries. We note that display cases and large objects are excluded from the simulations (white rectangular areas in Figure 2A). Clearly, this method quickly loses accuracy with increasing distance from the sensor. Sensor data interpolation is however useful in case a real-time control of the environmental conditions needs to be implemented, for example to eliminate condensation on the windows.

The second type of modeling comprises physics-based models, incorporating Computational Fluid Dynamics (CFD), which provide very detailed environmental conditions of the museum galleries. CFD modeling is the basis for microclimate models and it uses sensor readings from the wireless sensor network as input for prescribing boundary conditions. The CFD models also require a mesh of the physical space, i.e., the dimensions, the location of windows and doors, objects of art, etc. The CFD model implemented generates air flow and temperature distribution across the whole three dimensional space. Specific details of the CFD model, including the use of sensor data for prescribing boundary conditions, are provided in [45]. Here we note that the East wall of Late Gothic Hall displays a historic tapestry hung from the top. Temperature distributions resulting from the simulations along the wall could then be used as input data sets for tapestry modeling to understand fabric expansion under temperature and humidity variation in the gallery [46]. Having three-dimensional temperature distributions (Figure 2B) would also enable museum curators to pinpoint the most stable locations in the galleries (i.e., with fewest environmental fluctuations). Furthermore, CFD modeling would allow conducting other studies, such as analyses of the impact people (which can be represented as moving heat sources in the model) may have on micro-climatic conditions and works of art housed in the museum.

With the interpolation and the CFD models, it is possible to simulate important physical phenomena in the heat and mass transfer process, like natural convection (buoyancy effects). The latter is an important factor to take into account within the context of simulating heat and mass transfer inside buildings. Furthermore, a hierarchy of models of varying complexity can be included as part of the framework. For instance, in addition to temperature sensor data, a model may take relative humidity sensor data as input in order to simulate the distribution for the air moisture in a room, in addition to that of air and temperature [32,45].

In general, CFD modeling is computationally more intensive than statistical modeling, but CFD it is more accurate. In the museum context, CFD modeling is useful for planning installations of works of art and for achieving long term object preservation goals. Interpolation models, being faster but less accurate, are useful for continuous optimization and adjustments of the HVAC systems of the museum.

### 2.3. Museum Characteristics

The Cloisters has multiple galleries with tall ceilings, interconnected with open corridors. The museum has large stained glass windows and several doors that directly connect most of the galleries to the outside environment. The physical characteristics of the museum or building envelope, i.e., its walls, windows, doors, and roof, affect the heat loss rate of the building.

**Figure 3:**
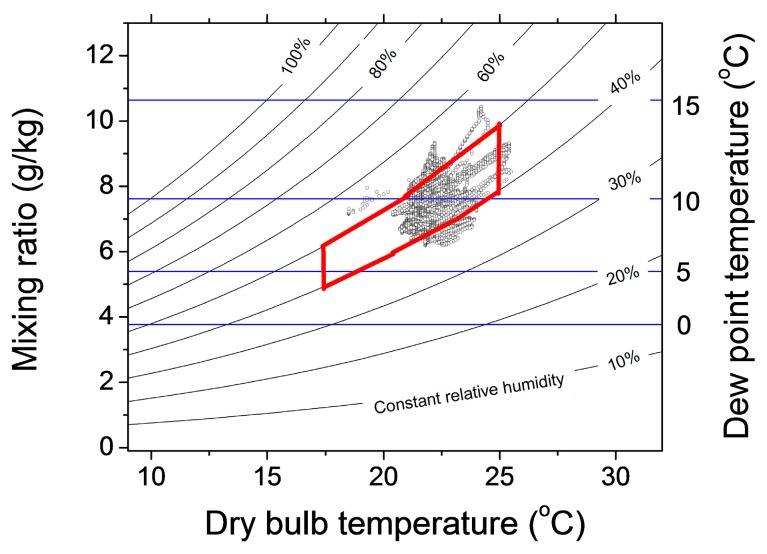
Psychrometric chart of the temperature and dew point measurements from all the sensors across a whole year period. More than 99% of the data points falls within the recommended operating region (red polygon).

Psychrometric charts are commonly used by building managers to quantify the stability of the environmental conditions using the dew point and air mixing ratio as a measure of the moisture in the air. The dew point quantifies the amount of moisture in the air, while the mixing ratio measures the amount of water (expressed in grams) in one kg of dry air. Psychrometric charts are usually plotted with data from an extended period of time, in order to capture seasonal changes. In this study, we aggregated the data from all sensors (Figure 3) across a one year period to demonstrate the very stable environmental conditions within the monitored galleries [47]. There are more than 1 million data points plotted in the image and only less than 1% of them are outside of the recommended range. The negligible number of outlier points in the psychrometric charts indicates the very stable environmental conditions in the galleries.

**Figure 4:**
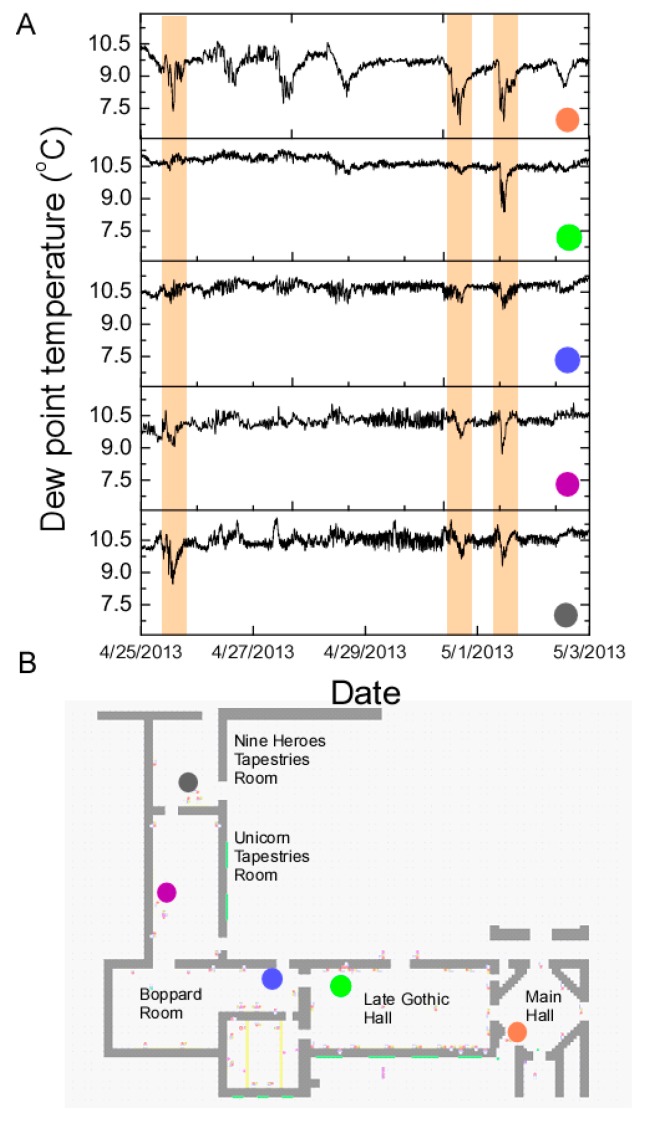
Correlated changes in dew point (**A**) across five galleries (**B**), where similar shape and timing in events can be traced. The events are triggered by opened doors or visitors entering in the museum.

Simultaneous monitoring of the environment in multiple galleries and entrance doors status (open/close) enables one to detect air circulation from the main entrance to other galleries. The dew point gradient changes across multiple galleries in response to a draft from an open door. One such case is shown in Figure 4, where the dew point drop in the main entrance halls is propagated throughout the galleries. Three of such events are highlighted by the red vertical bands across Figure 4A. If the galleries are farther situated from the main entrance, the changes in dew point are smaller (Figure 4B). The data was acquired from the period 26 April to 6 May 2013. We note that not all changes observed in the Main Hall can be detected in other galleries. While some of the galleries doors are open during the summer to the inner garden of the Cloister, the temperature and relative humidity remain very stable in the galleries demonstrating the HVAC system can compensate for change in temperature and relative humidity. The HVAC system tended to compensate for changes when doors were opened or to instantaneous environmental fluctuations.

## 3. Results

### 3.1. Micro-Environmental Monitoring

One of the goals of this study has been to generate a continuous temperature and humidity data spanning a period of three years. Both the steady state conditions and instantaneous fluctuations were investigated in this study. A denser sensor placement, monitoring temperature and humidity, was used in the Late Gothic Hall (see Figure 1A). The interest in the Late Gothic Hall was motivated, on one hand due to its proximity to the main museum entrance, which makes it more likely to suffer from microclimatic fluctuations derived from the dynamics of public attendance. On the other hand, the particular architectural characteristics of the hall, with its high ceilings and adjustable shading upper window array facing east, made it a candidate for complex dynamics due to external temperature/humidity contributions from day illumination effects and public attendance at different times of the day.

**Figure 5:**
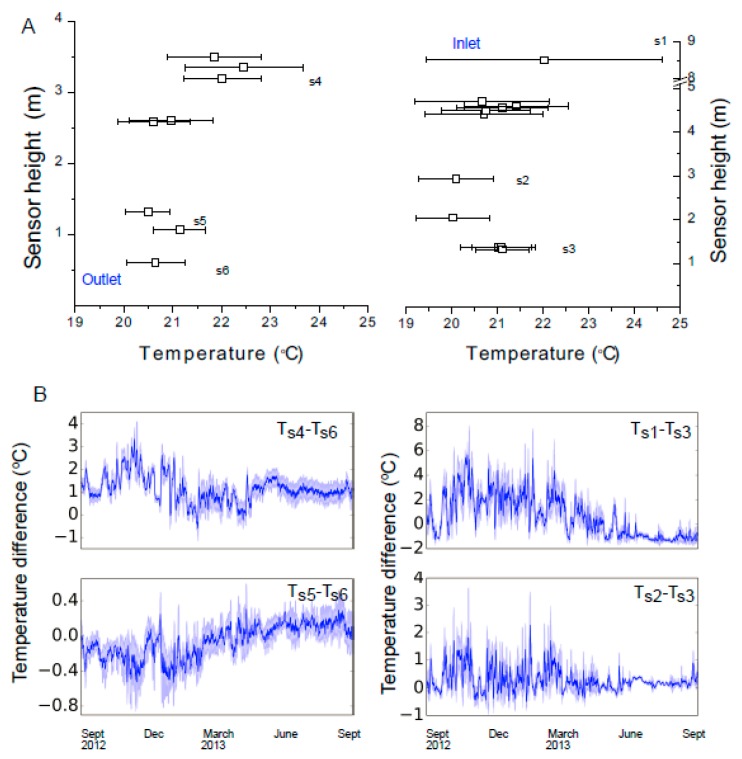
(**A**) Mean temperature and standard deviation distribution on the east (right graph) and west walls (left graph) of the Late Gothic Hall measured across a whole year for sensors positioned at different heights. (**B**) Temperature difference between pairs of sensors indicating seasonal trends in the environmental conditions.

The Late Gothic Hall heating/cooling system lets the air in at the upper level of the east wall while the outlet is on the lower end of the west wall. Within the gallery, a temperature stratification of less than 1 °C is observed as shown in Figure 5A where the mean and standard deviation of temperature for all sensors are plotted as function of sensor placement heights. All temperature sensors used by the wireless sensor nodes were calibrated at the beginning of the experiments by comparison with calibrated temperature sensors [32]. Furthermore the reading of the wireless sensors were compared with manual logging sensors used in the museum by curators during the time of the experiments. We note that the same HVAC system is used for cooling in the summer time and heating in the winter time and is maintaining temperature and relative humidity constant across different seasons. Higher variation as measured by standard deviation are detected at elevated heights (as mentioned above, the air inlet for the late Gothic Hall is at the upper level) while at the lower levels the temperature has less variations (most probably due to the fact that the temperature sensors used for HVAC control are situated at 0.5 m height). Figure 5B shows the temperature difference between sensor pairs measured across a year period (September 2012 until September 2013). The data is aggregated to daily value and plotted as daily value with ± one standard deviation as the band around the mean value. The temperature difference between the air inlet (top) and a bottom point on the Late Gothic’s Gallery East wall (T_s1_–T_s3_) has positive value during the winter time when the gallery is heated and is slightly negative during summer when the gallery is cooled. Sensors on the West wall show less temperature fluctuations as indicated in Figure 5A, most likely due to the fact that the feedback loop sensor used by the HVAC system is located on this wall.

Overall the temperature fluctuations are less pronounced at the lower levels of the gallery than at higher levels. As we previously discussed, the discrete sensor data are used in the CFD and interpolation models (Figure 2) to simulate the temperature at locations where sensors are not present (like the middle of the gallery). The sensor data combined with the simulations can be used for determining the most stable environmental condition in the gallery and use these data to guide art object placement in locations having lowest environmental stress.

**Figure 6:**
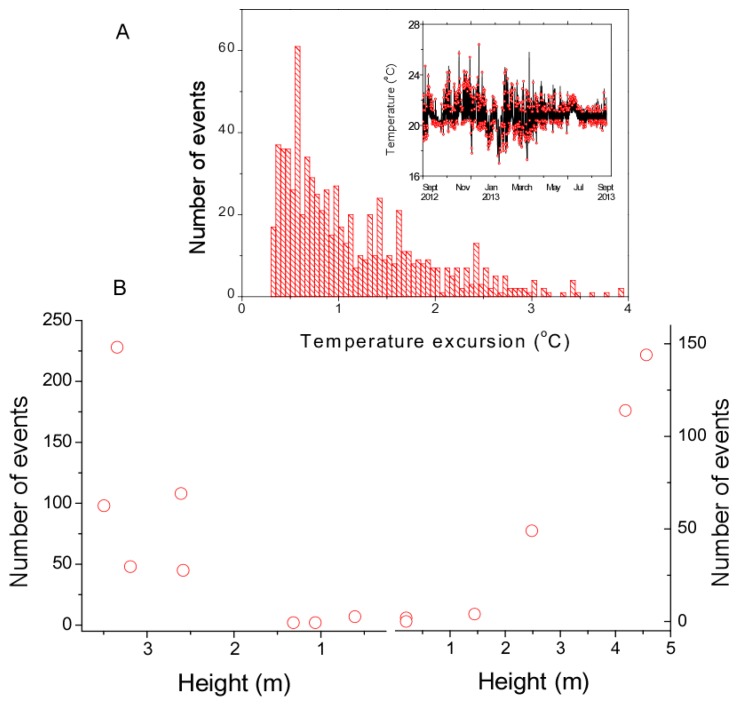
(**A**) Deviation of the temperature from its mean value plotted as a histogram for one year period and (**B**) the number of excursion events at different heights on the West (left image) and East wall (right image) of the Late Gothic Hall. The smallest changes in mean temperature is detected at the lower heights.

A temperature sensor trace across a whole year is shown as an inset in Figure 6A. The average HVAC set-point is fixed at around 20.1 °C and there are small fluctuations in temperature around the long term average. To quantify the stability of the temperature traces, a single event, called excursion, assumed when the temperature deviates more than 0.2 °C from the yearlong average temperature.

These excursions are shown as red dots on the temperature trace in Figure 6A. All the events are aggregated, binned, and plotted as a histogram (Figure 6A). This histogram shows the frequency of events when the temperature deviated from its long term medium value. As can be seen from Figure 6A, the 0.5 °C degree deviations were the most common.

In addition, as the deviation degree increased, the number of detected events decreased very quickly. Only a couple of events with temperature changes larger than a degree were detected during the whole year period. These deviations are shown in Figure 6B, where the left graph is for sensors positioned on the west wall while the right graph shows the sensors positioned on the east wall. In general, these results demonstrate the large dominance of the HVAC control in terms of temperature stability near the floor and in proximity to the air outlet grids. Some temperature stratification and larger fluctuations are observed closer to the ceilings. The increased number of temperature excursions may be a subject of consideration by the conservators for ascertaining the relevance of these stratified dependence fluctuations on wood objects placed at higher levels [3,9,34,48,49].

**Figure 7:**
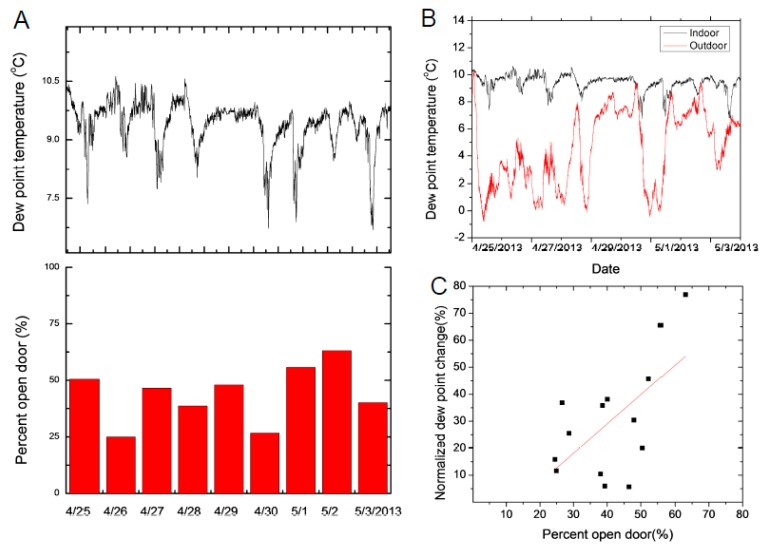
(**A**) The outdoor and (**B**) indoor dew point variation at the Main Hall when the entrance door is open indicates the high impact the outdoor air has on the indoor dew point changes. The correlation is more significant (27, 28 April and 1, 3 May) when the differences between indoor and outdoor dew points is more pronounced. (**C**) The normalized dew point change correlates with daily percent of entrance door kept open.

The MMT sensing platform can test the time scale of the capability of the HVAC system to address outside weather changes. Because of the temperature dependence of the relative humidity values, a careful analysis of moisture content [50] is advisable in view of the role it plays in the stability of many art pieces [2,25]. The highest variations in humidity and temperature are expected in the Main Hall (entrance) and consequently the Late Gothic Hall since is connected to the Main Hall through a passageway (see Figure 1A). The main impact of the indoor dew point variation can be attributed to the opening and to the closing of the entrance doors. The Main Hall has two entrance doors directly connecting the gallery to the outdoor (one door is mainly used by visiting groups), and, additionally, a stairway that is the main entrance to the museum by individual visitors. The stairway is also connected to the outdoor and tends to be drafty, bringing outdoor air into the Main Hall. For the door position sensing, a latch contact (reed) sensor was used that recorded whether the door was closed or open. The door position was sampled at 1 min intervals and the data was aggregated to a percentage of how much it was open during normal museum hours. The percentage was calculated as the total number of minutes the door was open divided by the number of minutes the museum is open for visitation. A snapshot of the change in dew point temperature versus the percentage of keeping the door open indicates that keeping the door open for extended time will decrease the dew point temperature in the Main Hall (Figure 7A). The change in indoor moisture level is mainly determined by outdoor moisture level and amount of exchange air with outdoor when the doors are open (Figure 7B). Thus both the outdoor dew point and the amount of time the entrance door is kept open will determine the indoor changes. The indoor and outdoor dew points (Figure 7B) indicate that the most significant indoor changes are detected when the differences between the indoor and outdoor dew point is largest.

The indoor dew point tracks the overall outdoor dew point trends (Figure 7B). Our data show a strong correlation when the outdoor dew point value is small but the correlation diminishes when the dew point gets around the average value (determined as the value during night time), indicating a good control of the average dew point to compensate for the air influx. This data set was acquired during the spring time when outdoor dew points tend to be smaller than indoor dew points in the museum. To quantify the change in dew point when an entrance door is open, we calculated the normalized dew point change that is defined as:

(1) $$\Delta T_{dew}=\frac{T_{dew,min}^{i}-T_{dew,mean}^{i}}{T_{dew,min}^{o}-T_{dew,min}^{i}}$$

where $T_{dew,min}^{i}$ is the daily minimum value of the indoor dew point while the entrance door is kept open, $T_{dew,mean}^{i}$ is the mean daily indoor dew point value, $T_{dew,min}^{o}$ in the daily minimum value of the outdoor dew point temperature.

When the normalized dew point change is compared with the daily percent open door value, the Pearson correlation coefficient is 0.62 (Figure 7C). Using a linear fit, a prediction can be made between the normalized dew point change and percentage the entrance door is kept open.

Obviously, changes in indoor relative humidity can be detected when the difference between the outdoor and indoor relative humidity is relatively large. The most important impact for environmental changes is the high humidity and temperature conditions during summer/autumn at The Cloisters. An influx of outside air due to the opening and closing of the doors leading to the internal garden or the entrance doors provides a source of air with large moisture content, which may lead to undesired condensation on cooler objects inside the halls.

**Figure 8:**
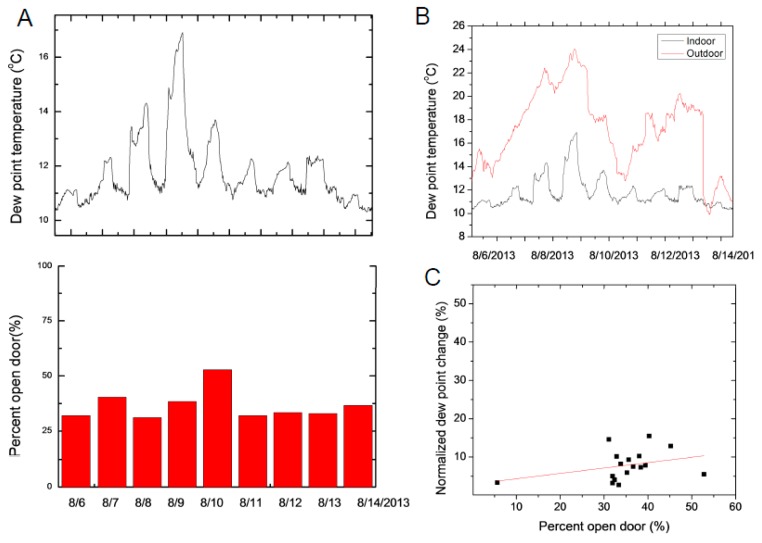
(**A**) Variation in dew point temperature change and the percentage the visitor door is kept open in the main hall. The door is kept open more over the weekend (8 and 9 August), creating the largest change in dew point. (**B**) The change in the indoor dew point is largest when outdoor dew point is highest and (**C**) very weak correlation between normalized dew point change and the amount of time entrance door is open during this time period.

A scenario when the outdoor air has higher moisture content than the indoor air is shown in Figure 8. The indoor dew point increases when the outdoor air seeps into the Main Hall. The largest indoor moisture level change is detected when the differences between outdoor and indoor dew points are most significant (Figure 8B). In this case the normalized dew point change is calculated with the daily maximum values of the dew point rather than the minimum as was described in Equation (1). The Pearson correlation between the normalized dew point change and the percentage of open door is 0.34. The smaller correlation can be due to the impact of visitors that may generate additional changes and the operational settings of the HVAC system.

We note that when the indoor and outdoor dew points are similar in value, there is little change in the indoor dew point value despite that the entrance door is kept open. On 29 April 2013 (Figure 7B) and on 13 August 2013 (Figure 8B) the indoor dew point variations were insignificant.

### 3.2. Air Quality

Many of the cultural heritage objects in The Cloisters have metal embossed or incorporated in them (for example silver fibers in tapestries) or are purely made out of metals (coins, statues). The prevalent way in which metal objects decay is by corrosion [51]. For corrosion to be triggered there are two components that have to occur simultaneously: the presence of a water monolayer on the surface of metallic objects and gaseous contamination molecules that can be absorbed/diffused on its surface. Once the gaseous contamination is absorbed on the surface, it interacts electrochemically with the metal atoms and transforms them in corrosion byproduct. Many metals are selective in its interaction with different gaseous contaminations. We note that corrosion-free environments can be achieved only through heavy air filtering and chemically scrubbing, but those solutions are often not financial feasible. Second order effects detected on top of very small corrosion signals can be further used to understand the connections between the building and the outdoor environments, as well as the impact of outdoor air quality on corrosion rates.

If the outdoor air is filtered before allowing it inside a museum, the impact of gaseous contamination on art objects can be minimal. Our air quality corrosion sensors indicate that indoor air quality at The Cloisters is very clean [51]. The air quality was investigated through silver and copper corrosion monitoring in the galleries. The corrosion sensors (Figure 1C) are exposed to the gallery environment in the Late Gothic Hall and the Unicorn Gallery and were also included inside the enclosures in the Boppard Room. Additionally, the sensors were exposed to the outdoor environment by placing them in the front on the door that opens to the Garden from the Late Gothic Hall. The door to the Garden is sometimes kept open during summer time, allowing visitors to move from the Late Gothic Hall to the Garden. When the door is opened during the summer outside air enters into the gallery.

**Figure 9:**
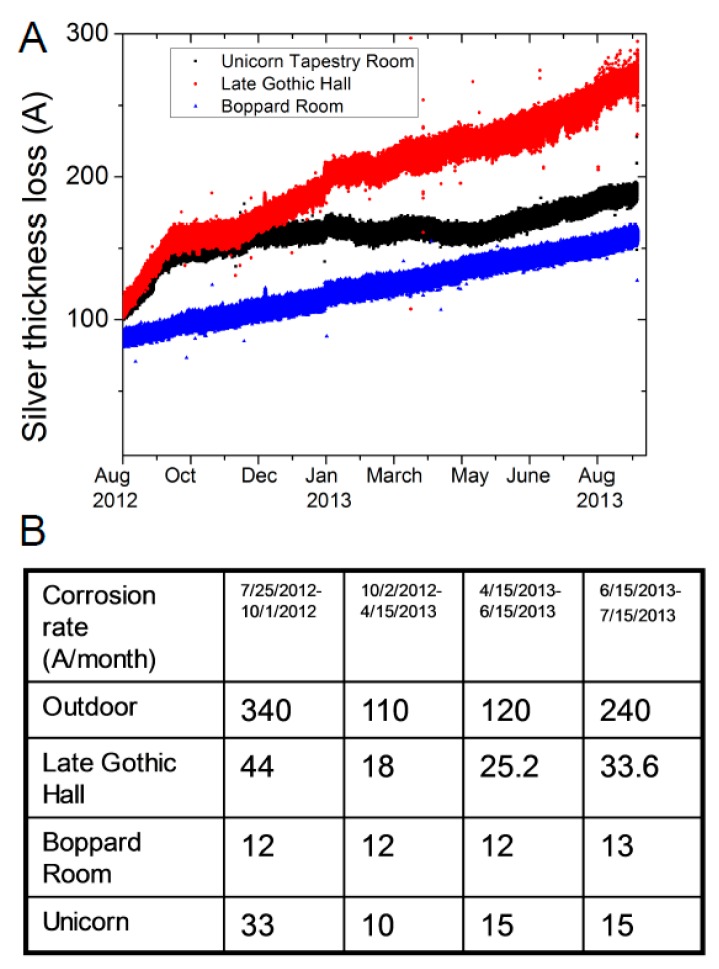
Change in silver film thickness (**A**) due to pollution molecules chemically modifying the film surface measured across a year period. The corrosion rate change (**B**) during 4 distinct time interval inside and outside the museum is shown in table B.

The corrosion sensors are resistively sensing a reduction of the total film thickness (Figure 9A) and the change in film thickness is converted to corrosion rate by calculating the rate of change across a time interval. Corrosion tends to have seasonal variations due to variations in pollutant concentration and climate. As a first step in corrosion rate calculations, the resistance value of the metal film is temperature compensated to eliminate the change in resistance of the metal film due to temperature fluctuations. The corrosion rate is calculated by relating current film thickness to the one month old average value of film thickness calculated between 45 and 15 days average from a given current time. Referencing to the monthly resistance value the current corrosion rate is estimated every 5 min. The average value of the corrosion rates is shown in the table in Figure 9B. The table has 4 distinct periods related to the time intervals when the protective panels around the arcades surrounding the middle garden are installed (winter time). The glass panels are raised to minimize the heat loss during winter and also to protect the architecture. For the time period of this study, the panels were raised on 1 October 2012 and were removed on 15 April 2013. When the panels are not mounted, the corrosion rates are higher as measured by the outdoor air quality sensors (first and last column in Figure 9B table). Once the panels are in place, the corrosion rates drop significantly (from 2 October 2012 until 15 April 2013), indicating that the panels act as an “air filter” in addition to insulating the galleries from outdoor weather conditions.

The same trend in corrosion rate is observed for the indoor corrosion sensors. The values are high during autumn (first column in Figure 9B table) and then their values drop significantly for the next time period (panels installed) and increase when the panels are removed. The only sensors that shows no seasonal variations in the corrosion rate are the ones placed inside the protective cases (Boppard Room) indicating the effectiveness in using exhibition cases to protect metal objects.

The corrosion sensor data further validates that there is a weak air exchange between the galleries and the outdoor. When the outdoor corrosion rate is high, indoor corrosion rates increases and when the outdoor corrosion rate is low, then the indoor values are also low. The corrosion rate values presented in Figure 9B are the average value across the specified time period. The corrosion sensors do show daily variations on top of long term corrosion rates trends and the increased values are driven by air moisture levels (Figure 8A).

Multiple studies over the years have demonstrated that corrosion of metal surfaces can be accelerated by increased pollutant concentration under high humidity weather. Dose corrosion functions have been proposed to correlate indoor/outdoor corrosion rates to humidity, gaseous pollutant concentrations and temperature changes [28]. The main gaseous contaminations that accelerate silver and copper corrosion rates are: sulfur dioxide (SO_2_), nitrogen dioxide (NO_2_), and hydrogen sulfide (H_2_S). The above gaseous contaminants are mainly generated by industrial activities. The daily SO_2_ concentrations used in our analysis were obtained from a nearby air quality station, within 10 miles from the location of the museum. In addition, weather data was extracted both, from nearby weather stations operated by New York City and also from outdoor temperature and relative humidity measurements acquired by our wireless sensor network.

The corrosion rate (CR) was fitted based on a dose function [52]:

(2) $$\mathrm{CR}\propto \left({\mathrm{SO}}_{2}\right)\cdot e^{-\frac{E_{a}}{k_{B}T}}\cdot e^{\left(b\cdot \mathrm{RH}\right)}$$

where SO_2_ is the concentration of pollutant gas measured by a nearby weather station, *T* is temperature, and RH is the outdoor relative humidity. In the above formula k_B_ is Boltzmann constant and *E_a_* is the activation energy. In this study the two parameters that provide the best fitting (smallest standard deviation between measured and generated data) are $E_{a}=4.24\;\mathrm{eV}$ and b=0.035.

**Figure 10:**
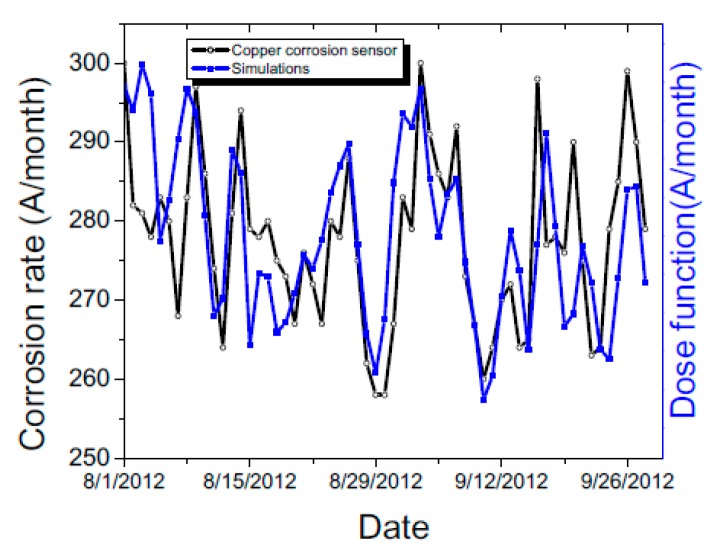
Measured outdoor corrosion rate and dose function modeling considering SO_2_ concentration and weather data from a nearby weather station.

The fitting of the corrosion rate as related to the modeling is shown in Figure 10. The change in outdoor corrosion rate can be very well reproduced using only SO_2_ concentration, temperature and relative humidity change. We note that the relative humidity change has the highest impact on the outdoor corrosion rate modeling while gaseous concentrations being the second most important dependent parameter. The values of the dose function developed for this section is a guide to predict corrosion levels based on weather and air quality data. Since weather forecast and seasonal pollution trends can be easily established for many locations based on historical and real time measurements, the corrosion rate can be forecasted days in advance to better guide preservation and conservation.

A similar fitting for indoor corrosion rate modeling is hard to quantify as the temperature and relative humidity are very stable inside the galleries and there are no measurements available for the indoor gaseous contaminations levels.

### 3.3. Impact of Visitor Flow

Visitor presence can influence the microclimate in a space and it is most easily detectable in small galleries or rooms. Every visitor will locally increase the temperature and will change the relative humidity; a human body will emit the energy corresponding to an electrical light bulb (~350 kJ/h). While the temperature and relative humidity change impact is tiny in many cases it can be measured with high sensitivity sensors.

A study of microclimate change due to visitor presence was carried out in the Campin Room (see Figure 1A) where people presence sensor and environmental sensors are time synchronized. Two infrared sensors were mounted at the ceiling—these would count the number of people in the room across an area of 5 m by 5 m at every 1 min interval.

**Figure 11:**
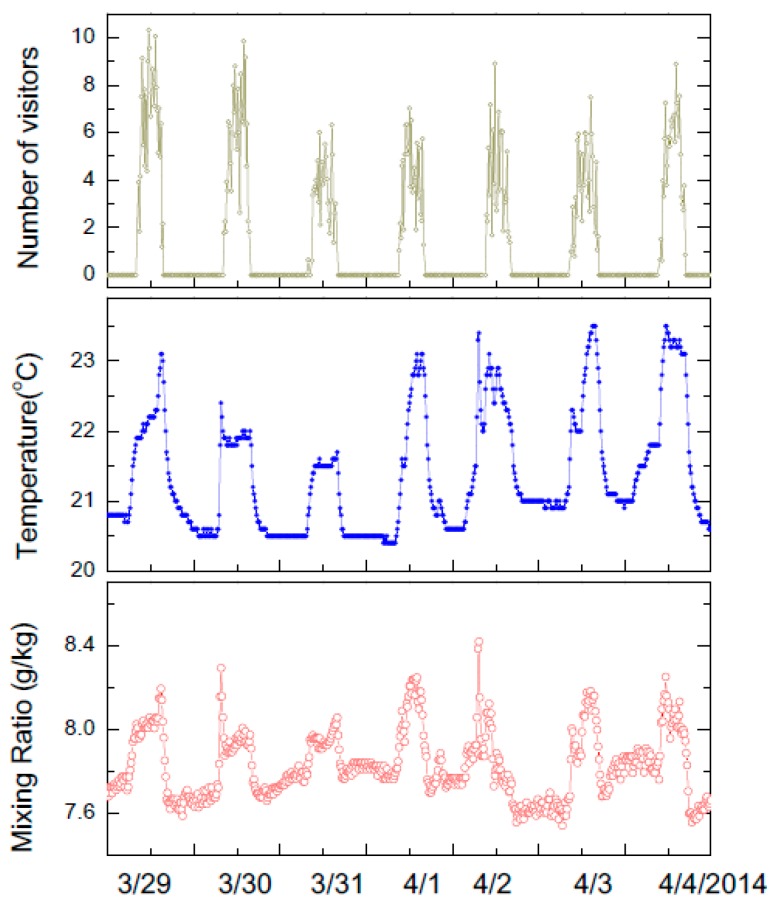
Visitor induced change in temperature and mixing ratio during the museum open hours. On average, a number of 10 visitors change the temperature by 1.5 °C and create an extra 1 gram of moisture.

The visitor presence sensor indicates that room occupation is low during the morning and late afternoon but increased to 8 to 10 visitors in early afternoon. As visitors entered the gallery, the temperature and moisture level increases (Figure 11). Typically the visitors are spending around 15 min in the gallery.

The microclimate change was assessed by calculating the air mixing ratio change due to visitor presence. This mixing ratio describes the additional amount of moisture created in the gallery as the number of visitors in the galleries increased. The change was around 0.7 g of additional moisture when around 10 people are present in the room. We note that the temperature of the inlet air stream did not change during visiting hours, further indicating that changes were due to the presence of people in the room. The change in temperature measured nearby paintings situated at face level was around 0.1 °C, whereas the overall change in temperature was 1.5 °C at the ceiling level.

The correlation between the change in temperature and mixing ratio indicates the importance of time synchronized measurements acquired at high temporal frequencies (Figure 11). These changes can be much localized in space and time. Also, we note that such data sets can be used to minimize environmental fluctuations by guiding in a controlled way visitors in galleries such that changes are minimal. Furthermore, proactive precooling of a room can be carried out based on the expected visitor numbers that would enter the gallery, the expected time they will remain in the galleries, and readjustment of the environmental conditions.

### 3.4. Dew Point and Window Condensation

Condensation may be encountered in museums where the constant indoor temperature and relative humidity settings can lead to the situation where inside glass temperature is lower than the ambient dew points [50]. The condensed water on windows, collected in the troughs at the bottom of each window, can pose risks to the medieval tapestry displayed right underneath the windows, in addition to contributing to the long term damage to the window. Estimating the occurrence of condensation as well as long term object preservation requires collecting microclimatic information through extended periods of time.

At The Cloisters, protective glazing with IR and UV filters was installed to protect the medieval glass windows and stained glass installed at the outer perimeter of the building from weathering, as well as to provide an adequate envelope to the collection. In the Late Gothic Hall, however, the relatively recent installation of a double glazing system has not prevented the formation of extensive condensation on the inner surface of the protective glazing [53] during winter, implying high humidity in the glass interspace. While the diamond pane windows are historic elements dating from 1938, they are installed in a medieval stone surrounding which can also be damaged by the migration of moisture. Moreover, condensed water on windows, collected in the troughs at the bottom of each window, can pose risks to the medieval tapestry displayed right underneath the windows. Estimating the occurrence of condensation using the climatic data collected by wireless sensor network has thus been paramount in protecting these objects.

**Figure 12:**
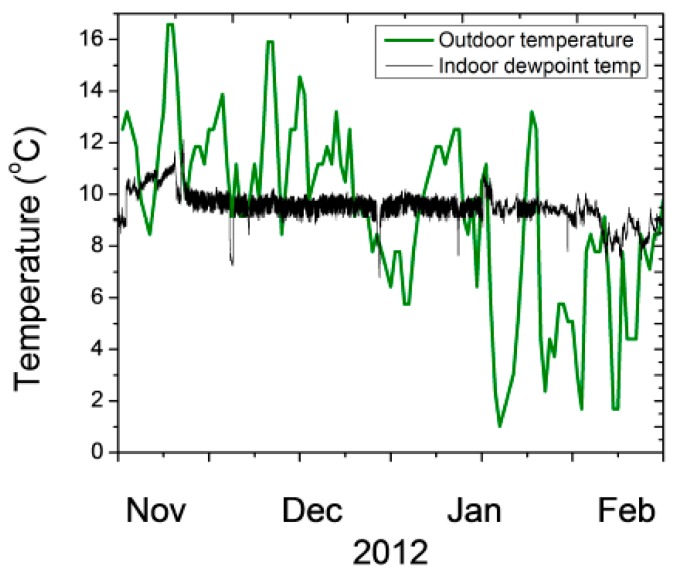
Modeling the time of year when window condensation may occur on a single pane window. When the outdoor temperature drops below indoor dew point temperature water film formation is very likely on the windows.

For a single pane window with historical glass, the heat transfer across the glass can be modeled using well documented parameters that translate the outdoor temperature to the glass temperature facing the galleries [53]. One important factor that could remediate potential damage from condensation is to develop analytical tools to predict condensation and try to prevent it. There are two important elements required for condensation prediction. One is short term weather forecasting for next few days and a window condensation model that translates outdoor temperature values to the window surface temperature facing the gallery. Figure 12 shows the time periods when the outdoor temperature may fall below the indoor dew point and condensation may occur on the window. The inner surface of the window, taking into consideration heat transfer, is 2.6 °C higher than the outdoor temperature.

Outdoor weather forecast is available five days in advance at hourly time intervals. Condensation risk assessment takes into account the forecast to predict when indoor dew point may (assumed constant based on historical trends) be higher than outdoor temperature. We note that forecast is updated daily so the longer term weather forecast beyond a day may be less relevant. Data is used to validate the accuracy of model by observing condensation and compare it with prediction. Window condensation forecasting could be used to adjust heating and or temperature to prevent condensation. Window condensation forecasting was implemented in autumn 2013 based on the wireless sensor network data to predict the days when water may form on the window surface. Nevertheless, protecting the windows with additional panels to better insulate them is under consideration by the curators to reduce the risk of condensation.

## 4. Discussion

Current standards for environmental parameters in the museums recommend a tight temperature and relative humidity ranges. These standards were established more than 70 years ago and are followed as industry best practices. In many buildings the environmental conditions are measured by one or a few sensors that may not be located in the ideal measurement place (traditionally the sensors are attached to a wall in close proximity of air inlet/outlets). Deploying dense wireless sensing has the advantage to assess the environmental conditions, across the whole building, in all three dimensions and understand air stratification, the environmental connections between rooms and the insulating properties of the building walls, windows and doors.

A good understanding of the microclimates that may form in large galleries or exhibition rooms have the potential to enhance the utilization of the galleries and determine the best location where art objects may be placed with minimized risk. The same principle of microclimates can be easily extended to hospitals, schools, museums, convention centers and eldercare facilities where air quality and fluctuations in the environmental conditions can have a significant impact on health and wellbeing of people.

There is a need to develop comprehensive platforms that combine sensor data with statistical and physical modeling and contextual information about building layout, building construction material that allow easy access to the data and can be used both as a real time monitoring tool as well as a repository of long term trends. Ultimately these platforms need to be combined with risk models for art objects that can estimate the impact environment on art object in a realistic environment rather than laboratory experiments [24]. Such efforts should rely on wireless sensor network which can be easily placed and rearranged in these buildings to comprehensively model the environment in areas where sensors cannot be easily placed [10,54]. This work is an attempt to establish such baseline sensing and modeling technique where analytics can be used in real time to adjust environmental conditions in response to visitor presence or outdoor air environmental conditions.

## 5. Conclusions

The environments in many museums are maintained very stable by setting very tight temperature and relative humidity ranges. There is an ongoing effort to assess the impact of expanding the temperature and relative humidity beyond the current acceptable and recommended ranges. Before such environmental changes may be accepted there is a clear need to assess by extensive studies the impact of changing environment on art objects. Risk models that quantify the temperature and relative humidity fluctuations on art objects are developed by museum curators and scientist. All these models will require data feeds to quantify in real time the impact of micro environment and display these models output along with the microclimate conditions. Platforms that integrate data and models need to have scalability included to extend these risk studies across large geographies and climates. Dense wireless sensing monitoring system can measure the microclimate conditions in galleries and feed the data to a range of models (physics-based and statistical) to estimate temporal variations and fluctuations of the temperature and air moisture levels between sensing points. Correlation studies and statistical analysis can quantify microclimate fluctuations as the number of visitors in the galleries are changing and/or outdoor air may enter in the galleries. This study demonstrated that the environmental conditions at The Cloisters are extremely stable and the microclimate data can be leveraged to guide curators in identifying the most stable locations in galleries and guide them in better arranging exhibits. One advantage of the wireless sensor network is the possibility to measure data and to couple the same wireless radios to transducers attached to exhibited object to understand how they react to environmental fluctuations. This type of monitoring can be used to compare the objects’ response when they are loaned to other exhibition and track the long term exposure of objects to different climates.
